# Supplementary material for: Light/Dark and Temperature Cycling Modulate Metabolic Electron Flow in Pseudomonas aeruginosa Biofilms
Source: mBio. 2022 Aug 8;13(4):e01407-22. doi: 10.1128/mbio.01407-22 (PMC9426528; doi:10.1128/mbio.01407-22)
Supplement: FIG S1 [file mbio.01407-22-s0001.pdf]

A

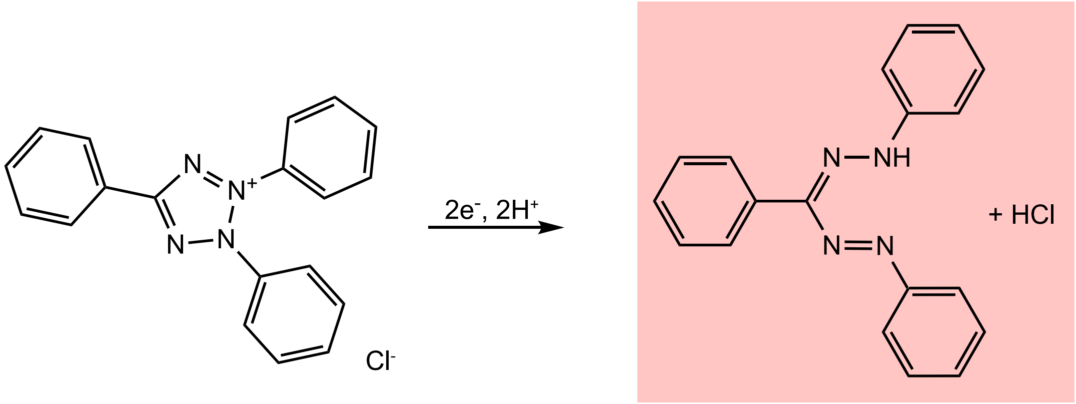

2,3,5-triphenyltetrazolium chloride

1,3,5-triphenylformazan

B

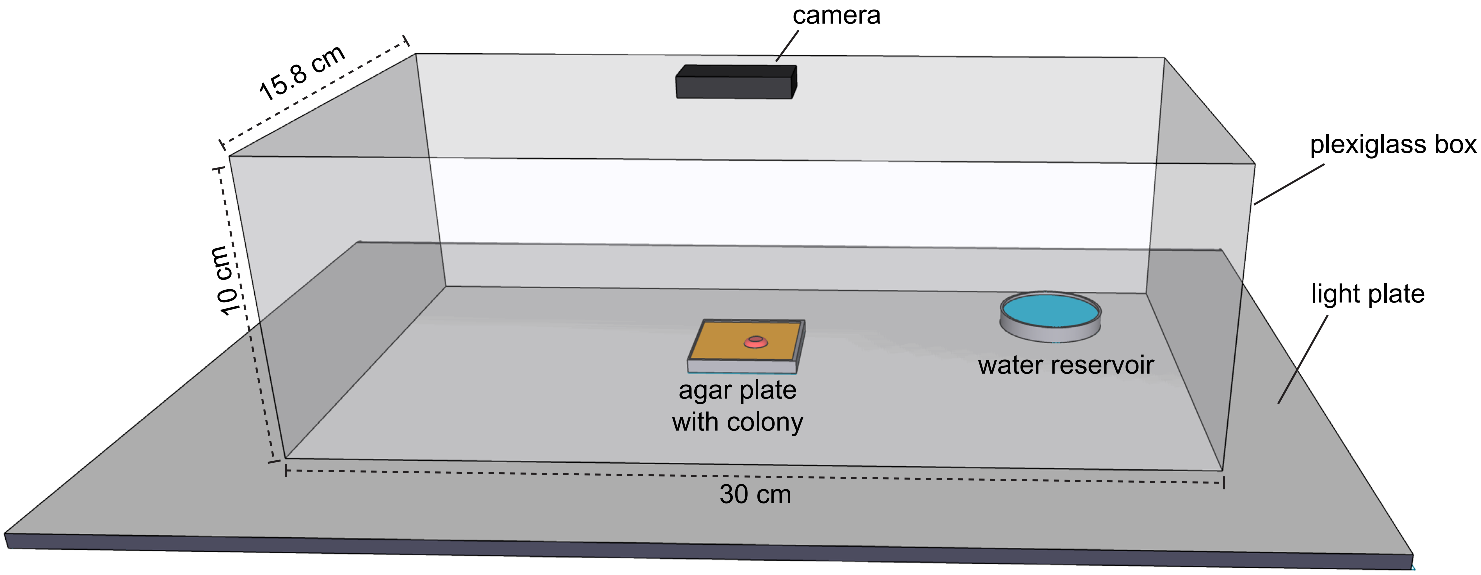

Figure S1. (A) Schematic showing the structure and reduction of TTC. **(B)** Schematic of incubation setup used to generate time-lapse movies of colony biofilm growth and TTC reduction.
