# Supplementary material for: Light/Dark and Temperature Cycling Modulate Metabolic Electron Flow in Pseudomonas aeruginosa Biofilms
Source: mBio. 2022 Aug 8;13(4):e01407-22. doi: 10.1128/mbio.01407-22 (PMC9426528; doi:10.1128/mbio.01407-22)
Supplement: FIG S2 [file mbio.01407-22-s0002.pdf]

**Supplemental Figure 2**

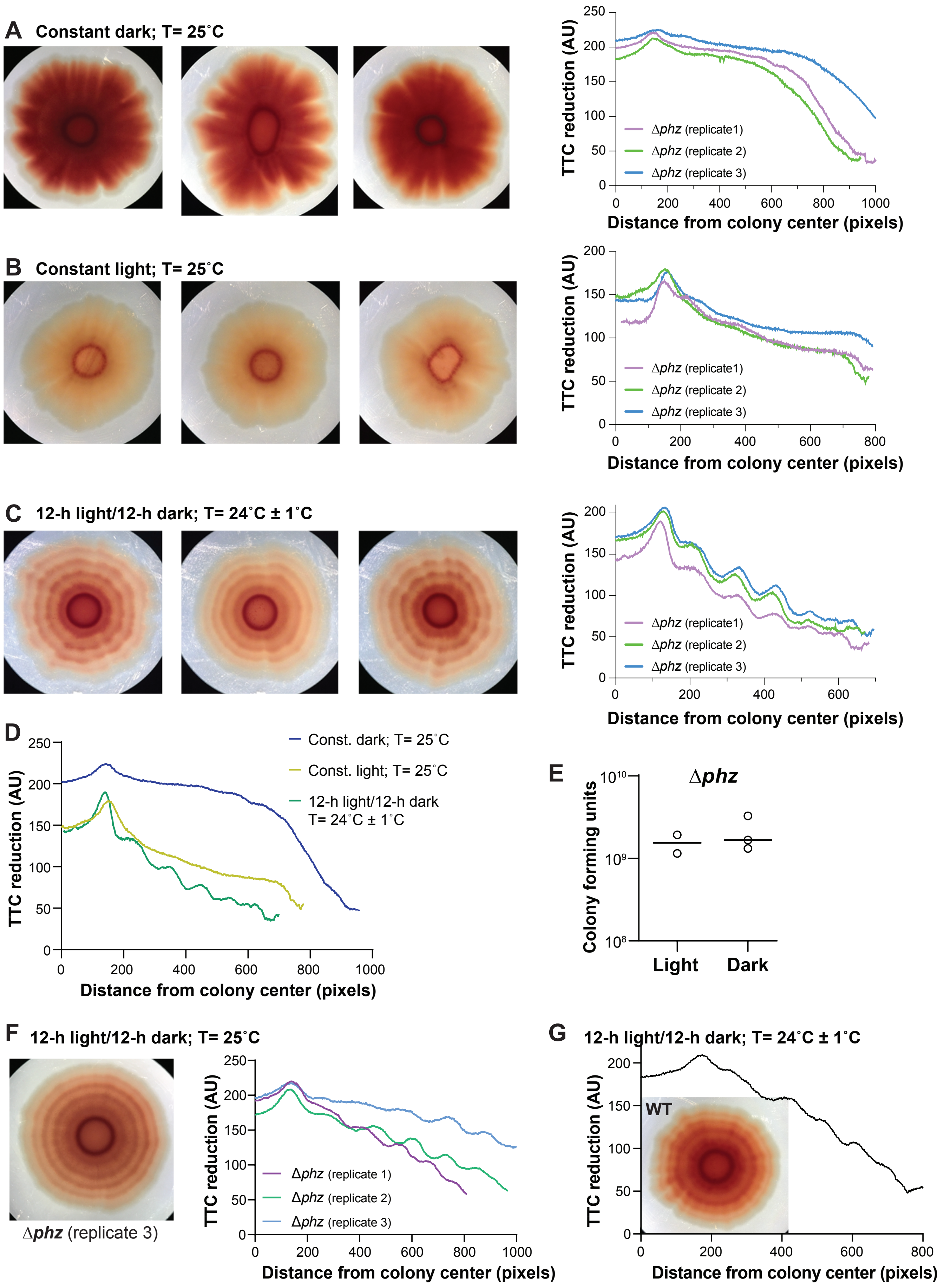

**Figure S2. (A-C)** Left: Replicates of Δ*phz* biofilms grown in constant dark at 25°C (A), in constant light at 25°C (B), and light and temperature cycling, 12-h light 25°C, 12-h dark 23°C (C). Right: Quantification of TTC reduction. **(D)** Comparison of representative TTC reduction quantification from all three conditions in panels A-C. **(E)** Colony forming units for Δ*phz* biofilms grown in constant light or constant dark at 25°C for 4 days. **(F)** Left: Representative Δ*phz* biofilm grown in light cycling (12-h light, 12-h dark) at constant temperature. Right: Quantification of TTC reduction of three replicates. **(G)** Quantification of TTC reduction in WT biofilm (inset) grown in light and temperature cycling, 12-h light 25°C, 12-h dark 23°C.
