# Supplementary material for: Light/Dark and Temperature Cycling Modulate Metabolic Electron Flow in Pseudomonas aeruginosa Biofilms
Source: mBio. 2022 Aug 8;13(4):e01407-22. doi: 10.1128/mbio.01407-22 (PMC9426528; doi:10.1128/mbio.01407-22)
Supplement: FIG S3 [file mbio.01407-22-s0003.pdf]

Temperature cycling

Supplemental Figure 3

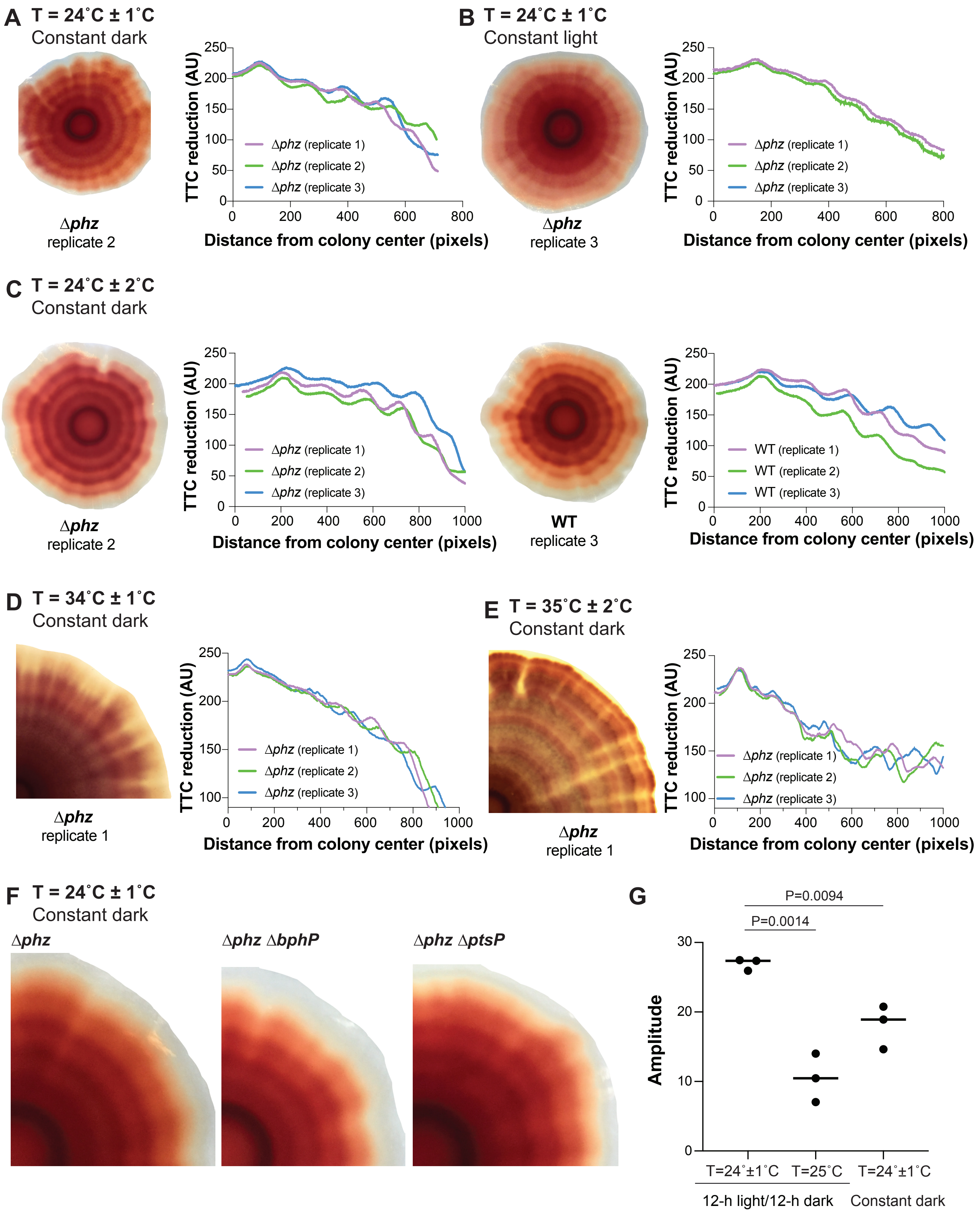

Figure S3. (A) Left: Representative replicate for Figure 1F. Right: Quantification of red color intensity (i.e., TTC reduction) at the indicated distance from the biofilm center for a radius of the biofilm for three replicates. **(B)** Left: Representative replicate for Δ*phz* biofilm grown in constant light with 24 °C +/- 1°C temperature cycling. Right: Quantification of red color intensity (i.e., TTC reduction) at the indicated distance from the biofilm center for a radius of the biofilm for three replicates. **(C)** Left: Representative WT or Δ*phz* biofilm grown in constant dark with 24°C +/- 2°C temperature cycling. Right: Quantification of TTC reduction for three replicates per condition. **(D&E)** Representative replicates for Δ*phz* biofilms grown in constant dark with 34°C +/- 1°C and 35°C +/- 2°C temperature cycling. **(F)** Light sensor mutants grown in constant dark with 24°C +/- 1°C temperature cycling, on medium containing 0.004% TTC. (**G**) Amplitudes of changes in TTC reduction for colonies grown in light and temperature cycling conditions are significantly higher than those for colonies grown in only light or temperature cycling conditions. Amplitudes were derived from detrended data in **Figures S2C, S2F and S3A**. Unpaired t-tests were performed using GraphPad Prism 9.4.0.
