# Supplementary material for: Light/Dark and Temperature Cycling Modulate Metabolic Electron Flow in Pseudomonas aeruginosa Biofilms
Source: mBio. 2022 Aug 8;13(4):e01407-22. doi: 10.1128/mbio.01407-22 (PMC9426528; doi:10.1128/mbio.01407-22)
Supplement: FIG S4 [file mbio.01407-22-s0004.pdf]

Supplemental Figure 4

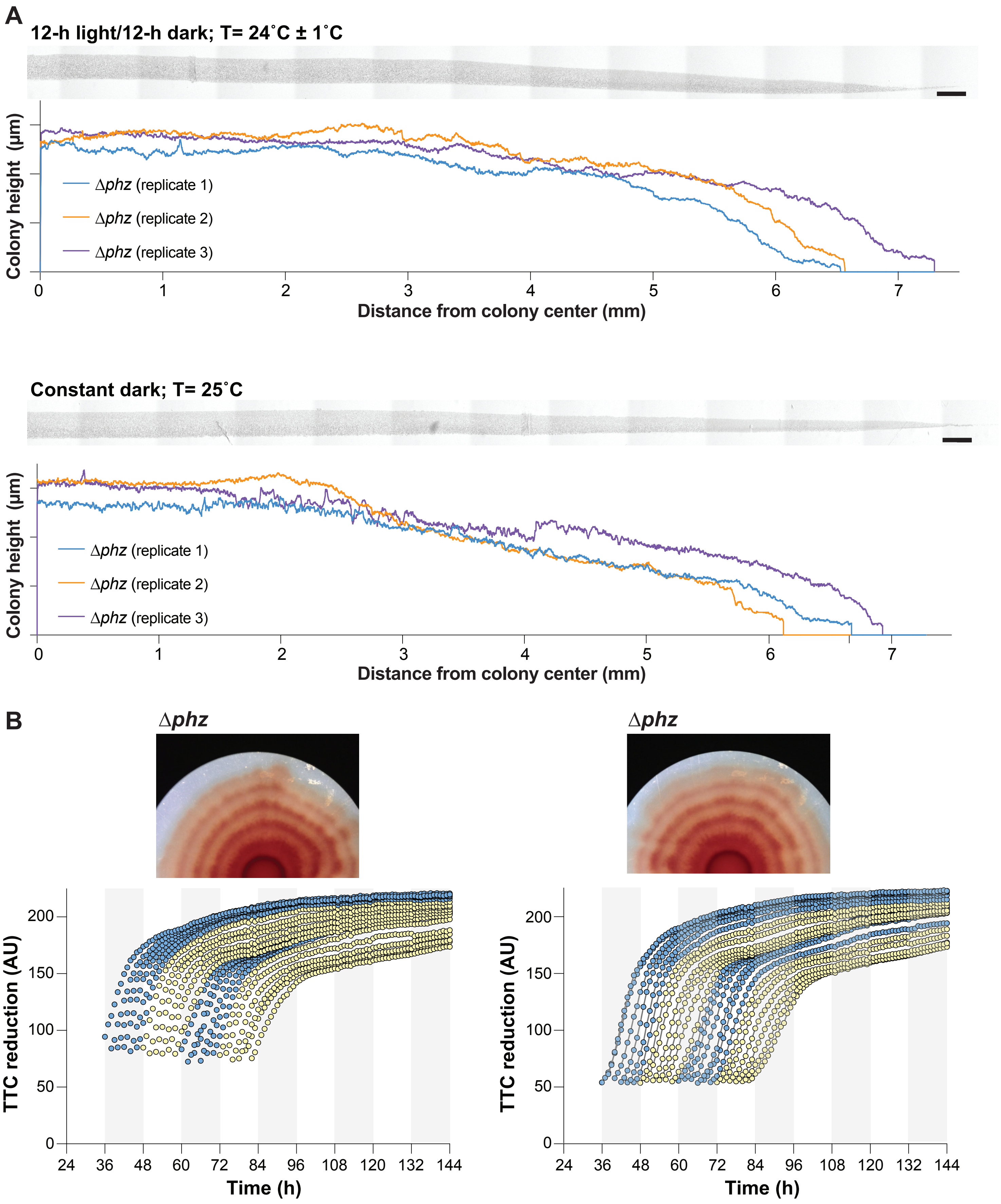

Figure S4. (A) Representative thin sections of Δ*phz* biofilms grown on medium containing 0.004% TTC for 3 days. Top: Section of biofilm grown with L/D and 24 ± 1 °C cycling. Bottom: Section of biofilm grown in the dark and at 25°C. Scale bars are 200 μm. For each condition, three replicates were quantified and graphed. Biological triplicates are shown. **(B)** Replicates for figure 2B. Traces corresponding to biomass appearing during dark intervals are shown in shades of blue; those corresponding to biomass appearing during light intervals are shown in shades of yellow.
