# Supplementary material for: Light/Dark and Temperature Cycling Modulate Metabolic Electron Flow in Pseudomonas aeruginosa Biofilms
Source: mBio. 2022 Aug 8;13(4):e01407-22. doi: 10.1128/mbio.01407-22 (PMC9426528; doi:10.1128/mbio.01407-22)
Supplement: TABLE S1 [file mbio.01407-22-s0007.pdf]

**Supplemental Table 1A: Strains used in This study**				
Pseudomonas aeruginosa strains				
**Strain**	**Number**	**Description**	**Source**	**Used in figure**
PA14 Δ*phz*	LD24	PA14 with deletions in *phzA1-G1* (*PA14_09480-09410*) and *phzA2-G2* (*PA14_39970-39880*) operons	(1)	1C-G, 2B, 2C, 3, 4, 5, 6A-C(top)
PA14 Δ*phz* Δ*pel*	LD83	PA14 Δ*phz* with deletions in genes *pelB-G* (*PA14_24490-24560*)	(2)	3A, 3B
PA14 Δ*phz* Δ*bphP*	LD4480	PA14 Δ*phz* with deletion in the bacteriophytochrome gene *bphP* (*PA14_10700*)	This study	4
PA14 Δ*phz* Δ*ptsP*	LD3131	PA14 Δ*phz* with deletion in the phosphoenolpyruvate-protein transferase gene *ptsP* (*PA14_04410*)	This study	4
PA14 Δ*phz* Δ*cco1/2*	LD1938	PA14 Δ*phz* with deletions of both *cco* operons (*PA14_44340-44400*)	(3)	2D, 6C(mid)
PA14 Δ*phz* ΔPa*Cco1*	LD2104	PA14 Δ*phz* with deletions in the *cox* operon (*PA14_01290-01320*), the *cyo* operon (*PA14_47150-47210*), the *cio* operon (*PA14_13030-13040*) and the *cco2* operon (*PA14_44340-44360*)	This study	6C(bot)
Escherichia coli strains				
UQ950	LD44	E. coli DH5 λ*pir* strain for cloning. F- Δ(*argF-lac*)169φ80 *dlacZ58*(Δ*M15*) *glnV44*(AS) *rfbD1 gyrA96*(Nal^R) *recA1 endA1 spoT thi-1 hsdR17 deoR λpir*$^+$	D. Lies	
BW29427	LD661	Donor strain for conjugation. thrB1004 pro thi rpsL hsdS lacZ ΔM15RP4-1360 Δ(araBAD)567 ΔdapA1314::[erm pir(wt)]	W. Metcalf, University of Illinois	
S17-1	LD2901	Str^R , Tp^R , F− RP4-2-Tc::Mu *aphA*::Tn7 *recA* λpir lysogen	(6)	
Saccharomyces cerevisiae strains				
InvSc1	LD676	MATa/MATalpha leu2/leu2 trp1-289/ trp1-289 ura3-52/ ura3-52 his3-Δ1/his3-Δ1	This study	
Pseudomonas aeruginosa strains used in screen for putative light sensors				
**Strain**	**Number**	**Description**	**Source**	**Transposon plate location**
Δ*PA14_38740*	LD2594	PA14 with deletion in the two-component sensor *PA14_38740*	This study	N/A
Δ*pilS*	LD2612	PA14 with deletion in the two-component sensor gene *pilS* (*PA14_60250*)	This study	N/A
Δ*phz* Δ*prpR*	LD2593	PA14 Δ*phz* with deletion in *prpR* (*PA14_36000*)	This study	N/A

Δ*phz* Δ*pilS*	LD2613	PA14 Δ*phz* with deletion in the two-component sensor gene *pilS* (*PA14_60250*)	This study	N/A
Δ*PA14_46860-46840*	LD696	PA14 with deletion in *PA14_46860*, *PA14_46850*, and *PA14_46840*	This study	N/A
Δ*PA14_38970-38990*	LD2271	PA14 with deletion in the two-component sensor gene *PA14_38970* and *PA14_38990*	This study	N/A
Δ*PA14_66320*	LD291	PA14 with deletion in *PA14_66320*	(4)	N/A
Δ*phz* Δ*PA14_38740*	LD2595	PA14 Δ*phz* with deletion in the two-component sensor gene *PA14_38740*	(4)	N/A
Δ*phz* Δ*PA14_66320*	LD290	PA14 Δ*phz* with deletion in *PA14_66320*	(4)	N/A
Δ*PA14_40210*	LD2610	PA14 with deletion in *PA14_40210*	This study	N/A
Δ*ntrB*	LD2594	PA14 with deletion in *ntrB* (*PA14_67670*)	This study	N/A
Δ*phz* Δ*PA14_38970-38990*	LD2272	PA14 Δ*phz* with deletion in the two-component sensor gene *PA14_38970* and *PA14_38990*	This study	N/A
ΔPA14_04420	LD2077	PA14 with deletion in *PA14_04420*	This study	N/A
Δ*PA14_46850*	LD694	PA14 with deletion in the transcriptional regulator *PA14_04420*	This study	N/A
Δ*phz* Δ*PA14_40210*	LD2611	PA14 Δ*phz* with deletion in *PA14_40210*	This study	N/A
Δ*rmcA*	LD2227	PA14 with deletion in *rmcA* (*PA14_07500*)	(4)	N/A
Δ*PA14_48830*	LD1788	PA14 with deletion in the transcriptional regulator *PA14_48830*	This study	N/A
Δ*phz* Δ*PA14_04420*	LD2078	PA14 Δ*phz* with deletion in *PA14_04420*	This study	N/A
Δ*phz* Δ*PA14_48830*	LD1113	PA14 Δ*phz* with deletion in the transcriptional regulator *PA14_48830*	This study	N/A
Δ*PA14_36000*	LD2592	PA14 with deletion in *prpR* (*PA14_36000*)	This study	N/A
Δ*PA14_57170*	LD1338	PA14 with deletions in the two component sensor gene *PA14_57170*	This study	N/A
Δ*phz* Δ*rmcA*	LD2228	PA14 Δ*phz* with deletion in *rmcA* (*PA14_07500*)	(4)	N/A
Δ*phz* Δ*PA14_57170*	LD1339	PA14 Δ*phz* with deletions in the two component sensor gene *PA14_57170*	This study	N/A
PA14_02220::Tn		PA14 with a MAR2xT7 transposon insertion in the *PA14_02220* gene	(5)	PAMr_nr_mas_11_3, G7
PA14_10770::Tn		PA14 with a MAR2xT7 transposon insertion in the *PA14_10770* gene	(5)	PAMr_nr_mas_08_1, B5
PA14_21700::Tn		PA14 with a MAR2xT7 transposon insertion in the *PA14_21700* gene	(5)	PAMr_nr_mas_15_2, B12

PA14_39560::Tn		PA14 with a MAR2xT7 transposon insertion in the *PA14_39560* gene	(5)	PAMr_nr_mas_6_3, B8
PA14_48160::Tn		PA14 with a MAR2xT7 transposon insertion in the *PA14_48160* gene	(5)	PAMr_nr_mas_9_1, H11
PA14_53140::Tn		PA14 with a MAR2xT7 transposon insertion in the *PA14_53140* gene	(5)	PAMr_nr_15_2, F3
PA14_59800::Tn		PA14 with a MAR2xT7 transposon insertion in the *PA14_59800* gene	(5)	PAMr_nr_mas_05_1, G11
PA14_65540::Tn	LD1812	PA14 with a MAR2xT7 transposon insertion in the *PA14_65540* gene	(5)	PAMr_nr_mas_03_2, C3
PA14_03720::Tn		PA14 with a MAR2xT7transposon insertion in the *PA14_03720* gene	(5)	PAMr_nr_mas_04_3, D7
PA14_10700::Tn		PA14 with a MAR2xT7 transposon insertion in the *PA14_10700* gene	(5)	PAMr_nr_mas_03_3, G2
PA14_24720::Tn		PA14 with a MAR2xT7 transposon insertion in the *PA14_24720* gene	(5)	PAMr_nr_mas_12_2, H12
PA14_39560::Tn		PA14 with a MAR2xT7 transposon insertion in the *PA14_39560* gene	(5)	PAMr_nr_mas_11_2, E10
PA14_49160::Tn		PA14 with a MAR2xT7 transposon insertion in the *PA14_49160* gene	(5)	PAMr_nr_mas_9_4, G9
PA14_53140::Tn		PA14 with a MAR2xT7 transposon insertion in the *PA14_53140* gene	(5)	PAMr_nr_mas_15_3, B4
PA14_59800::Tn		PA14 with a MAR2xT7 transposon insertion in the *PA14_59800* gene	(5)	PAMr_nr_mas_01_4, A5
PA14_65540::Tn	LD1813	PA14 with a MAR2xT7 transposon insertion in the *PA14_65540* gene	(5)	PAMr_nr_mas_07_4, B8
PA14_06950::Tn	LD1444	PA14 with a MAR2xT7 transposon insertion in the *PA14_06950* gene	(5)	ExMr_nr_mas_01_1, C10
PA14_11630::Tn		PA14 with a MAR2xT7 transposon insertion in the *PA14_11630* gene	(5)	PAMr_nr_mas_9_3, F9
PA14_32940::Tn		PA14 with a MAR2xT7 transposon insertion in the *PA14_32940* gene	(5)	PAMr_nr_mas_08_1, H7
PA14_44300::Tn		PA14 with a MAR2xT7 transposon insertion in the *PA14_44300* gene	(5)	PAMr_nr_mas_13_2, E11
PA14_50200::Tn	LD715	PA14 with a MAR2xT7 transposon insertion in the *PA14_50200* gene	(5)	PAMr_nr_mas_10_3, C5
PA14_53310::Tn		PA14 with a MAR2xT7 transposon insertion in the *PA14_53310* gene	(5)	PAMr_nr_mas_13_1, C3
PA14_60870::Tn		PA14 with a MAR2xT7 transposon insertion in the *PA14_60870* gene	(5)	PAMr_nr_mas_02_3, C10
PA14_70760::Tn		PA14 with a MAR2xT7 transposon insertion in the *PA14_70760* gene	(5)	PAMr_nr_mas_9_2, C1
PA14_07820::Tn		PA14 with a MAR2xT7 transposon insertion in the *PA14_07820* gene	(5)	PAMr_nr_mas_8_3, E1
PA14_11830::Tn		PA14 with a MAR2xT7 transposon insertion in the *PA14_11830* gene	(5)	PAMr_nr_mas_11_2, E4

PA14_36420::Tn		PA14 with a MAR2xT7 transposon insertion in the *PA14_36420* gene	(5)	PAMr_nr_mas _9_1, B8
PA14_46030::Tn		PA14 with a MAR2xT7 transposon insertion in the *PA14_46030* gene	(5)	PAMr_nr_mas _8_2, E3
PA14_50200::Tn		PA14 with a MAR2xT7 transposon insertion in the *PA14_50200* gene	(5)	PAMr_nr_mas _12_4, F12
PA14_53310::Tn		PA14 with a MAR2xT7 transposon insertion in the *PA14_53310* gene	(5)	PAMr_nr_mas _13_2, A3
PA14_61640::Tn		PA14 with a MAR2xT7 transposon insertion in the *PA14_61640* gene	(5)	PAMr_nr_mas _06_2, H4
PA14_71850::Tn		PA14 with a MAR2xT7 transposon insertion in the *PA14_71850* gene	(5)	PAMr_nr_mas _04_3, D11
PA14_09680::Tn		PA14 with a MAR2xT7 transposon insertion in the *PA14_09680* gene	(5)	PAMr_nr_mas _11_3, E10
PA14_12820::Tn		PA14 with a MAR2xT7 transposon insertion in the *PA14_12820* gene	(5)	PAMr_nr_mas _9_4, F2
PA14_37690::Tn		PA14 with a MAR2xT7 transposon insertion in the *PA14_37690* gene	(5)	PAMr_nr_mas _14_1, B2
PA14_46850::Tn	LD419	PA14 with a MAR2xT7 transposon insertion in the *PA14_46850* gene	(5)	PAMr_nr_mas _04_3, E6
PA14_52980::Tn	LD1445	PA14 with a MAR2xT7 transposon insertion in the *PA14_52980* gene	(5)	PAMr_nr_mas _01_3, D6
PA14_55780::Tn		PA14 with a MAR2xT7 transposon insertion in the *PA14_55780* gene	(5)	PAMr_nr_mas _11_4, B9
PA14_62530::Tn	LD411	PA14 with a MAR2xT7 transposon insertion in the *PA14_62530* gene	(5)	PAMr_nr_mas _06_2, A7
PA14_10290::Tn		PA14 with a MAR2xT7 transposon insertion in the *PA14_10290* gene	(5)	PAMr_nr_mas _05_4, D5
PA14_21700::Tn		PA14 with a MAR2xT7 transposon insertion in the *PA14_21700* gene	(5)	PAMr_nr_mas _07_2, C4
PA14_38570::Tn		PA14 with a MAR2xT7 transposon insertion in the *PA14_38570* gene	(5)	PAMr_nr_mas _11_2, H11
PA14_47910::Tn		PA14 with a MAR2xT7 transposon insertion in the *PA14_47910* gene	(5)	PAMr_nr_mas _03_4, E7
PA14_53140::Tn		PA14 with a MAR2xT7 transposon insertion in the *PA14_53140* gene	(5)	PAMr_nr_mas _10_4, B7
PA14_59780::Tn		PA14 with a MAR2xT7 transposon insertion in the *PA14_59780* gene	(5)	PAMr_nr_mas _15_1, G10
PA14_62530::Tn	LD412	PA14 with a MAR2xT7 transposon insertion in the *PA14_62530* gene	(5)	PAMr_nr_mas _15_1, F6
PA14_72390::Tn		PA14 with a MAR2xT7 transposon insertion in the *PA14_72390* gene	(5)	PAMr_nr_mas _07_1, D4
PA14_04410::Tn		PA14 with a MAR2xT7 transposon insertion in the *PA14_04410* gene	(5)	PAMr_nr_mas _14_3, A11
PA14_29620::Tn		PA14 with a MAR2xT7 transposon insertion in the *PA14_29620* gene	(5)	PAMr_nr_mas _01_2, E8

PA14_43350::Tn		PA14 with a MAR2xT7 transposon insertion in the *PA14_43350* gene	(5)	PAMr_nr_mas_05_2, A3
PA14_10190::Tn		PA14 with a MAR2xT7 transposon insertion in the *PA14_10190* gene	(5)	PAMr_nr_mas_08_4, F10
PA14_29620::Tn		PA14 with a MAR2xT7 transposon insertion in the *PA14_29620* gene	(5)	PAMr_nr_mas_10_4, G2
PA14_43350::Tn		PA14 with a MAR2xT7 transposon insertion in the *PA14_43350* gene	(5)	PAMr_nr_mas_12_4, H3
PA14_23190::Tn		PA14 with a MAR2xT7 transposon insertion in the *PA14_23190* gene	(5)	PAMr_nr_mas_11_4, G7
PA14_31330::Tn		PA14 with a MAR2xT7 transposon insertion in the *PA14_31330* gene	(5)	PAMr_nr_mas_03_2, B11
PA14_43430::Tn		PA14 with a MAR2xT7 transposon insertion in the *PA14_43430* gene	(5)	PAMr_nr_mas_09_3, D10
PA14_02910::Tn		PA14 with a MAR2xT7 transposon insertion in the *PA14_02910* gene	(5)	PAMr_nr_mas_05_2, A4
PA14_24510::Tn		PA14 with a MAR2xT7 transposon insertion in the *PA14_24510* gene	(5)	PAMr_nr_mas_09_4, B3
PA14_38500::Tn		PA14 with a MAR2xT7 transposon insertion in the *PA14_38500* gene	(5)	PAMr_nr_mas_12_3, B7
PA14_56430::Tn		PA14 with a MAR2xT7 transposon insertion in the *PA14_56430* gene	(5)	PAMr_nr_mas_13_3, E7
PA14_04410::Tn		PA14 with a MAR2xT7 transposon insertion in the *PA14_04410* gene	(5)	PAMr_nr_mas_05_2, G7
PA14_27570::Tn		PA14 with a MAR2xT7 transposon insertion in the *PA14_27570* gene	(5)	PAMr_nr_mas_02_1, C11
PA14_38500::Tn		PA14 with a MAR2xT7 transposon insertion in the *PA14_38500* gene	(5)	PAMr_nr_mas_15_3, E10
PA14_04410::Tn		PA14 with a MAR2xT7 transposon insertion in the *PA14_04410* gene	(5)	PAMr_nr_mas_07_2, G7
PA14_28070::Tn		PA14 with a MAR2xT7 transposon insertion in the *PA14_28070* gene	(5)	PAMr_nr_mas_06_4, F7
PA14_42970::Tn		PA14 with a MAR2xT7 transposon insertion in the *PA14_42970* gene	(5)	PAMr_nr_mas_04_3, H5

Supplemental Table 1B: Primers used in This study

**Primer number**	**Sequence**	**used to make plasmid number**
LD2475	aggcaaattctgttttatcagaccgcttctgcgttctgatCGGCTCGATCACTTCCTGCA	pLD3125
LD2476	ctgcgggttgtcgaaggtgagCATGGCTTCCTTGACCCGCTG	
LD2477	cagcgggtcaaggaagccatgCTCACCTTCGACAACCCGCAG	

LD2478	ggaattgtgagcggataacaatttcacacaggaaacagctGATGAACTCCTCGCCGCCC	
LD3030	ggaattgtgagcggataacaatttcacacaggaaacagctGCGCGGGGATGCCCATTTAT	pLD3618
LD3031	gttgcggttgcgctggttgtagttcgccagggtaaccgggg	
LD3032	ccccggttaccctggcgaactacaaccagcgcaaccgcaac	
LD3033	caaattctgttttatcagaccgcttctgcgttctgatCTCGTTCCGCGGAGTCGCCG	
LD1833	ccaggcaaattctgttttatcagaccgcttctgcgttctgatGGCGCGGTACTTTCACTC	pLD2622
LD1834	tgtccagcgtctcctgtatgtGGACTCCCATGGCTTCCT	
LD1835	aggaagccatgggagtccACATACAGGAGACGCTGGACA	
LD1836	ggaattgtgagcggataacaatttcacacaggaaacagctCTGCGGCTGACCCTCAGT	
LD1845	ccaggcaaattctgttttatcagaccgcttctgcgttctgatGAGTTCGCCCAGCTCACC	pLD2604
LD1846	ctggttgctctcgcagagttGGACCTGATCTTCCAGTTCG	
LD1847	cgaactggaagatcaggtccAACTCTGCGAGAGCAACCAG	
LD1848	ggaattgtgagcggataacaatttcacacaggaaacagctTTCTTCAGCTTCTCCTGTGC	
LD39	GGAATTGTGAGCGGATAACAATTTCACACAGGAAACAGCTGGTCGC GGATATAACCTGAA	pLD4558
LD40	CAGGTAGTCGATCAGTGCCGGACAAAGCTCGGAAAGACGA	
LD41	TCGTCTTTCCGAGCTTTGTCCGGCACTGATCGACTACCTG	
LD42	CCAGGCAAATTCTGTTTTATCAGACCGCTTCTGCGTTCTGACTACGA CATGGCGATCCTG	
LD1344	ccaggcaaattctgttttatcagaccgcttctgcgttctgatGACTTCGCCGCCTACCTG	pLD2267
LD1345	gaagtggtggcccaggacCGAGCTTGGCCATACCCTGAT	
LD1346	atcagggtatggccaagctcgGTCCTGGGCCACCACTTC	
LD1347	ggaattgtgagcggataacaatttcacacaggaaacagctGCTCGAACATCTTCCTCGAC	
LD1841	ccaggcaaattctgttttatcagaccgcttctgcgttctgatCTACCGGCCGATGTACTACC	pLD2624
LD1842	agagggtcatgcgggtccCAGGGACGAAGAAGGGAGAG	
LD1843	ctctcccttcttcgtccctgGGACCCGCATGACCCTCT	
LD1844	ggaattgtgagcggataacaatttcacacaggaaacagctGAAGATCACCGCCTACGACT	
LD1849	ccaggcaaattctgttttatcagaccgcttctgcgttctgatCTGGACGGCAAACCCTAC	pLD2605
LD1850	gctggctgatgatgttctggATGTACTCCAGGCGCAGTTC	
LD1851	gaactgcgcctggagtacatCCAGAACATCATCAGCCAGC	
LD1852	ggaattgtgagcggataacaatttcacacaggaaacagctCTTCAGCAGCTTGGGTTCC	
LD1059	GGAATTGTGAGCGGATAACAATTTCACACAGGAAACAGCTGCGAGC TGAGCAAGGGCCTG	pLD2079
LD1060	TCAGCAACAGGCCACGCAATGTAAGCGCCGCACGACGAAG	
LD1062	CTTCGTCGTGCGGCGCTTACATTGCGTGGCCTGTTGCTGA	

LD1061	CCAGGCAAATTCTGTTTTATCAGACCGCTTCTGCGTTCTGATGACGATGATCAGGCCGCCGC	
LD306	ggaattgtgagcggataacaatttcacacaggaaacagctCAGCAGCAGAACGAAACTCA	pLD1328
LD307	CTGGTTGGTGGACTGCCTGCGCAACCTGATAGAAGACG	
LD308	CGTCTTCTATCAGGTTGCGCAGGCAGTCCACCAACCAG	
LD309	ccaggcaaattctgttttatcagaccgcttctgcgttctgatCATGGTTCAGAAGCGCAGT	

Supplemental Table 1C: Plasmids used in This study

Number	Description	Source
pMQ30	7.5 kb mobilizable vector; oriT, sacB, GmR	(7)
pLD3125	Δ*ptsP* (Δ*PA14_04410*) PCR fragment introduced into pMQ30 by gap repair cloning in yeast strain InvSc1	This study
pLD3618	Δ*bphP* (Δ*PA14_10700*) PCR fragment introduced into pMQ30 by gap repair cloning in yeast strain InvSc1	This study
pLD2623	Δ*PA14_38740* (Δ*PA14_38740*) PCR fragment introduced into pMQ30 by gap repair cloning in yeast strain InvSc1	This study
pLD2604	Δ*PA14_60250* (Δ*PA14_60250*) PCR fragment introduced into pMQ30 by gap repair cloning in yeast strain InvSc1	This study
pLD2622	Δ*PA14_36000* (Δ*PA14_36000*) PCR fragment introduced into pMQ30 by gap repair cloning in yeast strain InvSc1	This study
pLD2627	Δ*PA14_38970-38990* (Δ*PA14_38970-38990*) PCR fragment introduced into pMQ30 by gap repair cloning in yeast strain InvSc1	This study
pLD216	Δ*PA14_66320* (Δ*PA14_66320*) PCR fragment introduced into pMQ30 by gap repair cloning in yeast strain InvSc1	(4)
pLD2623	Δ*PA14_38740* (Δ*PA14_38740*) PCR fragment introduced into pMQ30 by gap repair cloning in yeast strain InvSc1	(4)
pLD1308	Δ*PA14_03790* (Δ*PA14_03790*) PCR fragment introduced into pMQ30 by gap repair cloning in yeast strain InvSc1	(4)
pLD2624	Δ*PA14_40210* (Δ*PA14_40210*) PCR fragment introduced into pMQ30 by gap repair cloning in yeast strain InvSc1	This study
pLD2605	Δ*PA14_67670* (Δ*PA14_67670*) PCR fragment introduced into pMQ30 by gap repair cloning in yeast strain InvSc1	This study
pLD2079	Δ*PA14_04420* (Δ*PA14_04420*) PCR fragment introduced into pMQ30 by gap repair cloning in yeast strain InvSc1	This study
pLD909	Δ*rmcA* (Δ*PA14_07500*) PCR fragment introduced into pMQ30 by gap repair cloning in yeast strain InvSc1	(4)
pLD2079	Δ*PA14_04420* (Δ*PA14_04420*) PCR fragment introduced into pMQ30 by gap repair cloning in yeast strain InvSc1	(4)

SUPPLEMENTAL MATERIAL REFERENCES

1. Dietrich LEP, Price-Whelan A, Petersen A, Whiteley M, Newman DK. 2006. The phenazine pyocyanin is a terminal signalling factor in the quorum sensing network of *Pseudomonas aeruginosa*. Mol Microbiol 61:1308–1321.

2. Dietrich LEP, Okegbe C, Price-Whelan A, Sakhtah H, Hunter RC, Newman DK. 2013. Bacterial community morphogenesis is intimately linked to the intracellular redox state. J Bacteriol 195:1371–1380.

3. Jo J, Cortez KL, Cornell WC, Price-Whelan A, Dietrich LE. 2017. An orphan cbb3-type cytochrome oxidase subunit supports *Pseudomonas aeruginosa* biofilm growth and virulence. Elife 6:171538.

4. Okegbe C, Fields BL, Cole SJ, Beierschmitt C, Morgan CJ, Price-Whelan A, Stewart RC, Lee VT, Dietrich LEP. 2017. Electron-shuttling antibiotics structure bacterial communities by modulating cellular levels of c-di-GMP. Proc Natl Acad Sci U S A 114:E5236–E5245.

5. Liberati NT, Urbach JM, Miyata S, Lee DG, Drenkard E, Wu G, Villanueva J, Wei T, Ausubel FM. 2006. An ordered, nonredundant library of *Pseudomonas aeruginosa* strain PA14 transposon insertion mutants. Proc Natl Acad Sci U S A 103:2833–2838.

6. Simon R, Priefer U, Pühler A. 1983. A broad host range mobilization system for in vivo genetic engineering: Transposon mutagenesis in gram negative bacteria. Biotechnology 1:784–791.

7. Shanks RMQ, Caiazza NC, Hinsa SM, Toutain CM, O'Toole GA. 2006. *Saccharomyces cerevisiae*-based molecular tool kit for manipulation of genes from gram-negative bacteria. Appl Environ Microbiol 72:5027–5036.
