# Supplementary material for: Light/Dark and Temperature Cycling Modulate Metabolic Electron Flow in Pseudomonas aeruginosa Biofilms
Source: mBio. 2022 Aug 8;13(4):e01407-22. doi: 10.1128/mbio.01407-22 (PMC9426528; doi:10.1128/mbio.01407-22)
Supplement: FIG S5 [file mbio.01407-22-s0005.pdf]

A **Principal component analysis**

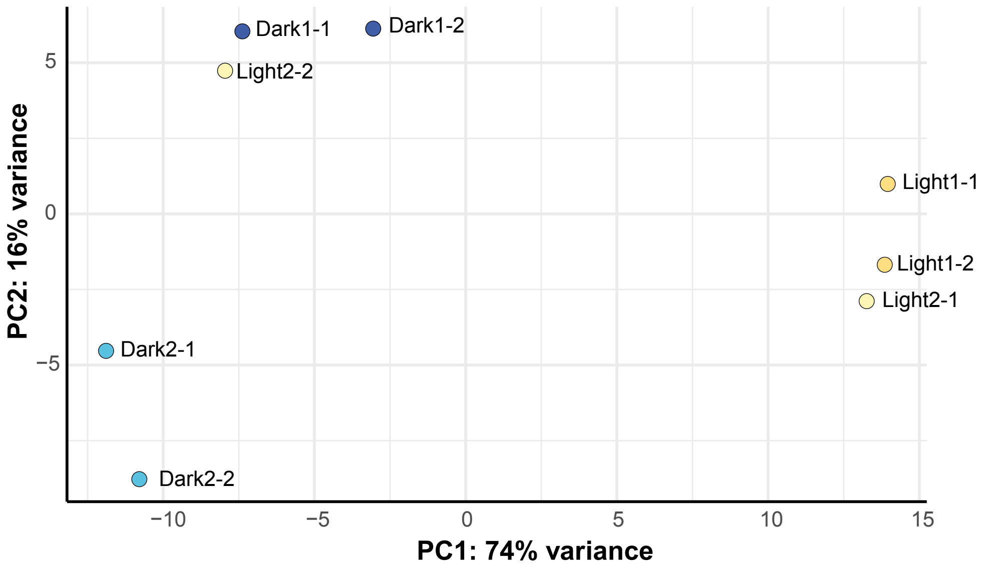

B

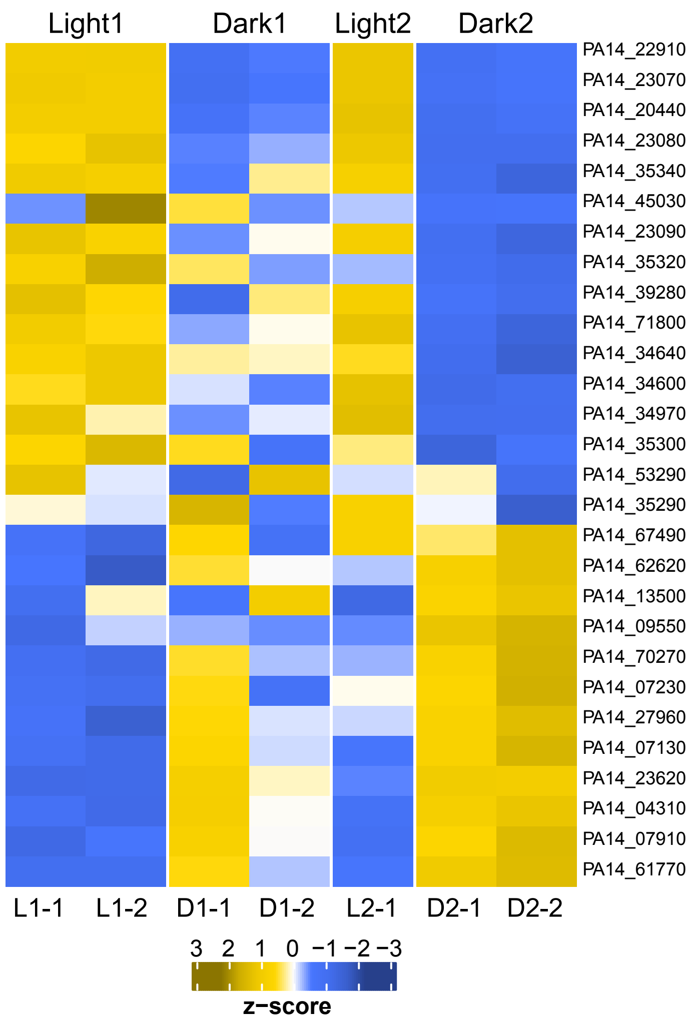

C

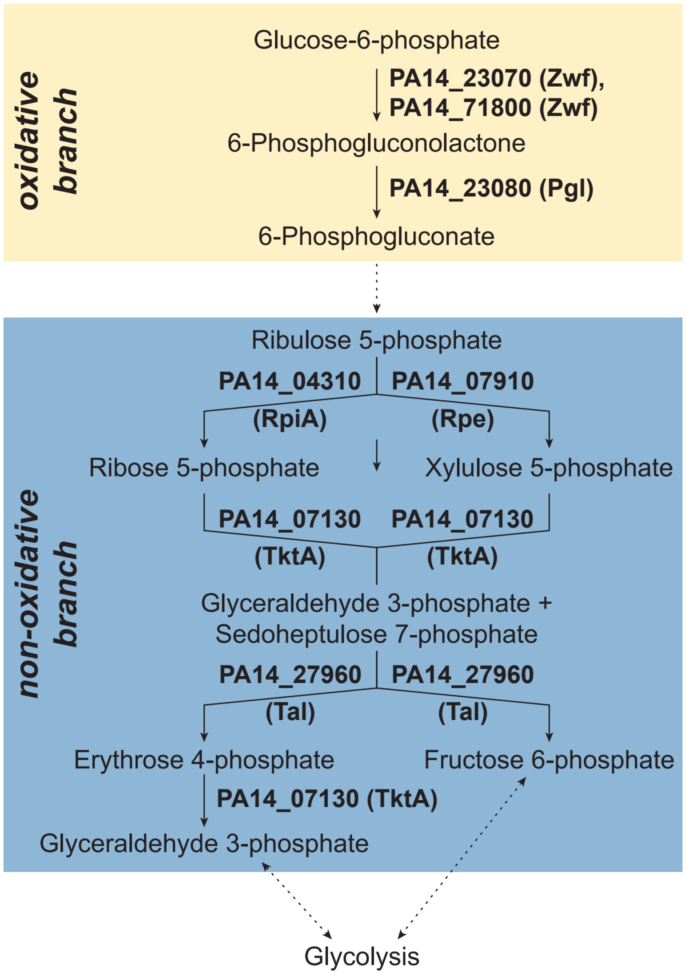

Figure S5. (A) Dimensionality reduction by principal component analysis was applied to RNAseq data for the full set of 8 samples obtained as shown in **Figure 6A**. Ninety percent of maximum possible variance was accounted for by the first two principle components PC1 and PC2. Each sample is plotted according to its values for PC1 and PC2. **(B)** Sixty-seven percent of genes involved in the pentose phosphate pathway were identified in the RNAseq as showing differential expression in biomass formed under light/dark and temperature cycling. Genes coding for enzymes involved in the oxidative branch were upregulated in biomass that formed under light/25 ˚C conditions, and genes coding for enzymes involved in the non-oxidative branch were upregulated in biomass that formed under dark/23˚C conditions. The heatmap shows genes involved in the pentose phosphate pathway, arranged according to their relative periodicity in samples from biomass formed under light/dark and temperature cycling. **(C)** Schematic of the pentose phosphate pathway highlighting selected genes whose expression was affected by growth under light/dark and temperature cycling conditions.
