# Supplementary material for: Light/Dark and Temperature Cycling Modulate Metabolic Electron Flow in Pseudomonas aeruginosa Biofilms
Source: mBio. 2022 Aug 8;13(4):e01407-22. doi: 10.1128/mbio.01407-22 (PMC9426528; doi:10.1128/mbio.01407-22)
Supplement: FIG S6 [file mbio.01407-22-s0006.pdf]

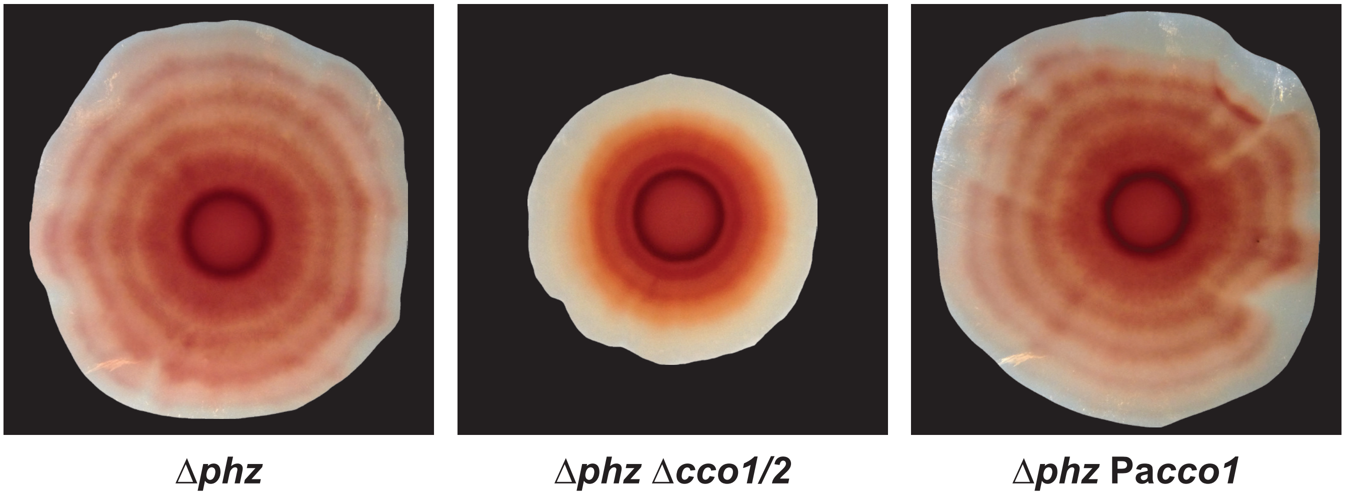

Figure S6. Images of Δ*phz*, Δ*phz*Δ*cco1cco2*, and Δ*phz* PaCco1 colony biofilms, corresponding to the analyses shown in Figure 7C and D.
